# Japanese cross-cultural validation study of the Pain Stage of Change Questionnaire

**DOI:** 10.1097/PR9.0000000000000711

**Published:** 2019-02-07

**Authors:** Tomonori Adachi, Momoka Sunohara, Kiyoka Enomoto, Keitaro Sasaki, Gaku Sakaue, Yoshitsugu Fujita, Yasuyuki Mizuno, Yoshiaki Okamoto, Kenji Miki, Masao Yukioka, Kazuhito Nitta, Narihito Iwashita, Hirotoshi Kitagawa, Masahiko Shibata, Jun Sasaki, Mark P. Jensen, Sei Fukui

**Affiliations:** a Pain Management Clinic, Shiga University of Medical Science Hospital, Otsu, Shiga, Japan; b Department of Rehabilitation Medicine, University of Washington, Seattle, WA, USA; c Japan Society for the Promotion of Science, Chiyoda, Tokyo, Japan; d Department of Psychology, Concordia University, Montreal, QC, Canada; e Center for Pain Management, Osaka University Medical Hospital, Suita, Osaka, Japan; f Plat Room Suita, Suita, Osaka, Japan; g Sakaue Clinic, Nishinomiya, Hyogo, Japan; h Department of Orthopedic Surgery, Japanese Red Cross Otsu Shiga Hospital, Otsu, Shiga, Japan; i Department of Psychosomatic and General Internal Medicine, Kansai Medical University, Hirakata, Osaka, Japan; j Department of Pharmacy, Ashiya Municipal Hospital, Ashiya, Hyogo, Japan; k Faculty of Health Science, Osaka Yukioka College of Health Science, Ibaraki, Osaka, Japan; l Center for Pain Management, Hayaishi Hospital, Osaka, Osaka, Japan; m Department of Rheumatology, Yukioka Hospital, Osaka, Osaka, Japan; n Nitta Pain Clinic, Toyonaka, Osaka, Japan; o Department of Anesthesiology, Shiga University of Medical Science, Otsu, Shiga, Japan; p Department of Health Science, Naragakuen University, Nara, Japan; q Department of Human Sciences, Graduate School of Human Sciences, Osaka University, Suita, Osaka, Japan

**Keywords:** Readiness to change, Motivation, Chronic pain, Cross-cultural validation

## Abstract

**Introduction:**

Although evidence supports efficacy of treatments that enhance self-management of chronic pain, the efficacy of these treatments has been hypothesized to be influenced by patient readiness for self-management. The Pain Stage of Change Questionnaire (PSOCQ) is a reliable and valid measure of patient readiness to self-manage pain. However, there is not yet a Japanese version of the PSOCQ (PSOCQ-J), which limits our ability to evaluate the role of readiness for pain self-management in function and treatment response in Japanese patients with chronic pain.

**Objective:**

Here, we sought to develop the PSOCQ-J and evaluate its psychometric properties.

**Methods:**

We recruited 201 patients with chronic pain. The study participants were asked to complete the PSOCQ-J and other measures assessing pain severity, pain interference, catastrophizing, self-efficacy, and pain coping strategies.

**Results:**

The results supported a 4-factor structure of the PSOCQ-J. We also found good to excellent internal consistencies and good test–retest reliabilities for the 4 scales. The Precontemplation scale had weak to moderate positive correlations with measures of pain-related dysfunction and maladaptive coping. The Action and Maintenance scales had weak to moderate positive correlations with measures of self-efficacy and adaptive coping. The Contemplation scale had weak positive correlations with measures of pain interference and both adaptive and maladaptive coping.

**Conclusions:**

The PSOCQ-J demonstrated adequate psychometric properties in a sample of Japanese patients with chronic pain. This measure can be used to evaluate the role that readiness to self-manage pain may play in adjustment to chronic pain in Japanese pain populations.

## 1. Introduction

Evidence supports the efficacy of treatments that enhance pain self-management for improving patient function.^11,23,48^ However, researchers have also noted substantial treatment dropout and relapse rates, as well as low adherence to these treatments.^14,35,45,46^ Readiness to adopt a self-management approach is believed to be an important factor that may influence treatment dropout, relapse, and adherence.^9,18,24^

Readiness to change is a key concept of the Transtheoretical Model of Behavior Change (TTM).^36–38^ The TTM was developed to describe behavior change processes in problematic health behaviors, such as alcohol abuse and smoking. The TTM hypothesizes that there are specific stages in a process of behavior change, and that individuals proceed through these stages because they work toward behavior change goals. Kerns and Habib^24^ proposed a pain readiness to change (PRC) model, which is an adaptation of the TTM for understanding how patients make changes in pain self-management. The PRC model hypothesizes 4 stages of change: Precontemplation (no intention to self-manage pain), Contemplation (considering the adoption of pain self-management), Action (making changes in self-management behaviors), and Maintenance (ongoing use of pain self-management strategies). The PRC model hypothesizes that the stage of change is associated with commitment to treatments teaching self-management strategies.^24^ In line with this model, high precontemplation has been shown to predict high dropout rates in interdisciplinary treatments.^4,12,25^ Moreover, decrease in the precontemplation and increases in the Contemplation, Action, and Maintenance have been shown to be associated with increases in use of adaptive coping strategies and improved outcomes.^5,10,12,21^

To evaluate the utility of the PRC model, as well as to evaluate the role that readiness to self-manage pain may play in patient function and treatment response, a measure of readiness to self-manage pain with adequate psychometric properties is required. The Pain Stage of Change Questionnaire (PSOCQ)^26^ is a self-report measure designed to assess the extent to which individuals are considering adopting a self-management approach to pain. The PSOCQ consists of scales representing the 4 stages of change.

Previous research supports the reliability and validity of the PSOCQ. For example, the PSOCQ scales have demonstrated patterns of associations indicating positive associations between the Precontemplation scale and measures of negative outcomes, and negative associations between this scale and positive outcomes, while the Action and Maintenance scales evidence the opposite pattern of associations.^6,19,20,26,32^ With respect to the Contemplation scale, the evidence tends to show weak associations with pain-related variables.^6,19,20,26,32^ Research also shows negative associations between the Precontemplation and both the Action and Maintenance scales, and positive associations between the Action scale and both Contemplation and Maintenance scales.^6,13,19,26,41,42^

Although the PSOCQ has been translated into a number of languages,^31,32,41^ there is not yet a Japanese version. Given evidence supporting the efficacy of self-management approaches among Asians (including Japanese) with chronic pain,^15,40,49^ developing a Japanese version of the PSOCQ (PSOCQ-J) would be useful, to be able to better understand the role of self-management readiness in function and treatment response in these patients.

To address this need, we sought to translate and examine the psychometric properties of a PSOCQ-J. We hypothesized that the PSOCQ-J would evidence a 4-factor structure. We also hypothesized that the scales would evidence at least adequate internal consistency (ie, Cronbach's alphas ≥0.70)^27^ and at least adequate test–retest reliability (ie, intraclass correlation [ICC] coefficients ≥0.60).^7^ We also anticipated that the Precontemplation scale would be negatively associated with the Action and Maintenance scales, and that the Action scale would be positively associated with the Contemplation and Maintenance scales. With respect to construct validity, we hypothesized that the Precontemplation scale would evidence a pattern of positive associations with measures of pain-related dysfunction, and negative associations with measures of functional and adaptive coping, while the Action and Maintenance scales would show the opposite pattern. We also hypothesized weak associations between the Contemplation scale and measures of pain-related function and coping. We hypothesized that individuals who are participating in exercise would score lower on Precontemplation, and higher on Contemplation, Action, and Maintenance. Because analgesic medication use could potentially be viewed as both an active self-management strategy as well as a passive one, we predicted weak and nonsignificant associations between the PSOCQ-J scales and analgesic medication use.

## 2. Methods

### 2.1. Participants

The study participants were outpatients with chronic pain recruited from 5 medical facilities: 2 pain clinics at secondary and tertiary care hospitals, 2 orthopedic surgery units at secondary care hospitals, and a unit of psychosomatic medicine at a tertiary care hospital. Eligibility criteria were as follows: (1) history of pain lasting at least 3 months, (2) at least 20 years of age, and (3) ability to read and write Japanese. We excluded individuals who had evidence of psychological dysfunction (eg, medical records indicating a history of dementia, schizophrenia, or substance abuse), which might interfere with study participation.

### 2.2. Procedure

We first translated the English PSOCQ instructions and items into Japanese using standard translation procedures (see Study Measures section). Study participants were then invited to participate in the study when they came each facility for their routine clinic visits. We also invited participants who returned to the clinics (in 1–12 weeks) to complete a second PSOCQ-J, to be able to compute ICC coefficients. Data collection was performed from October 2016 to July 2017. The Institutional Review Boards at Shiga University of Medical Science Hospital and Osaka University Graduate School of Human Sciences approved the study procedures. We obtained written informed consent form each participant before data collection.

### 2.3. Study measures

#### 2.3.1. Readiness to change: Japanese version of the Pain Stage of Change Questionnaire

The PSOCQ-J has 30 items that assess the responder's readiness to self-manage their pain. Study participants are asked to rate to how much extent they agree with each statement of items on a 5-point scale from 1 (“strongly disagree”) to 5 (“strongly agree”). The original PSOCQ has 4 scales labeled Precontemplation, Contemplation, Action, and Maintenance. Each scale is scored as the mean ratings provided for the items on that scale. Thus, the scale scores can range from 1 to 5, with higher scores indicating more agreement to the items reflecting each readiness stage.

#### 2.3.2. Translation procedures

We used Beaton's recommendation for translating cross-culturally adapting the PSOCQ into Japanese.^3^ Specifically, 2 bilingual Japanese PhD candidates majoring clinical psychology first independently translated the original English language PSOCQ into Japanese. Next, these 2 initial translations were merged into a single translation by the initial translators in collaboration with a bilingual PhD-level Japanese psychologist. Any differences in the specific translations for the instruction and items were discussed and resolved through consensus. As a third step, the revised version was reviewed by a Japanese anesthesiologist. After this, a back translation (from Japanese back into English) of the instructions and items was completed by a bilingual Japanese native. The back translation was then reviewed by the original PSOCQ developer, who provided feedback on any changes needed to ensure that the instructions and items reflected their original meaning. The instructions and items were revised as needed and reviewed again until the developer approved the final version of the PSOCQ-J.

As a final step, we asked ten outpatients with chronic pain attending a primary care pain clinic (8 women and 2 men, average age ± SD was 67.55 ± 12.81 years) to evaluate the PSOCQ-J for understandability and appropriateness of phrasing. Eighteen items for understandability and 8 items for appropriateness of phrasing (60% and 27% of the items, respectively) were rated as problematic by at least one of these patients. However, the most times any one item (items 1, 15, and 16) was rated as problematic was 3 (ie, all items were rated as clear and understandable by 70% to 100% of these patients). Also, these items were rated as difficult to understand with respect to them reflecting pain self-management, not with respect to the translation. Thus, no additional changes in the instructions or any of the items were deemed necessary.

To evaluate the convergent and discriminant validity of the PSOCQ-J, we then asked participants to complete 4 measures assessing pain-related variables.

#### 2.3.3. Pain severity and pain interference

Pain severity and interference were assessed by the Japanese version of the Brief Pain Inventory (BPI-J). Both the 4-item Pain Severity scale and 7-item Pain Interference scale of the Brief Pain Inventory have a great deal of evidence supporting their reliability and validity in a variety of patient samples with pain.^8,44^ Previous research also supports reliability and validity of the BPI-J in Japanese patients with cancer pain.^47^ The BPI-J Pain Severity items ask respondents to rate their worst, least, and average pain severity over the last week, as well as their current pain severity on 0 (“no pain”) to 10 (“pain as bad as you can imagine”) numerical rating scales. The average of the responses to these 4 items is computed to create the Pain Severity scale score. In the current sample, the internal consistency (Cronbach's alpha) for this scale was excellent (0.90). The BPI-J Pain Interference items ask respondents to rate the extent to which pain has interfered with 7 different activities of daily living over the last week (general activity, walking, work, mood, enjoyment of life, relations with others, and sleep) on a 0 (“does not interfere”) to 10 (“completely interferes”) numerical rating scale. Responses to these items are then averaged into a total Pain Interference scale score. In the current sample, the internal consistency coefficient for the Pain Interference scale was excellent (0.91).

#### 2.3.4. Catastrophizing

Catastrophizing was assessed using the Japanese version of the 13-item Pain Catastrophizing Scale (PCS-J).^30,43^ The PCS-J has evidence supporting its reliability and validity in Japanese patients with chronic pain.^17^ Each item reflects a pain-related negative thought, and respondents are asked to rate the frequency that they have each thought when they experience pain on a 0 (“not at all”) to 4 (“all the time”) scale. The responses are summed to form a total scale score that ranges from 0 to 52, with higher scores indicating higher levels of catastrophizing. The internal consistency coefficient for the PCS-J in the current sample was excellent (0.91).

#### 2.3.5. Self-efficacy

The Japanese version of the 10-item Pain Self-Efficacy Questionnaire (PSEQ-J) was used to assess the degree of confidence the participants had to perform certain activities in daily life despite pain.^34^ The PSEQ-J has evidence supporting its reliability and validity in Japanese patients with chronic pain.^1^ With the PSEQ-J, respondents are asked to rate how confident they are on a 0 (“not at all confident”) to 6 (“completely confident”) scale. The total PSEQ-J score ranges from 0 to 60, with higher scores representing higher levels of self-efficacy in functioning despite pain. The internal consistency coefficient for the PSEQ-J in the current sample was excellent (0.95).

#### 2.3.6. Pain-related coping

A Japanese version of the Chronic Pain Coping Inventory (CPCI-J) was used to assess 9 types of coping, reflected by 9 scales: Guarding, Resting, Asking for Assistance, Exercise/Stretch, Relaxation, Task Persistence, Coping Self-Statements, Pacing, and Seeking Social Support.^22^ The former 3 and the latter 6 strategies are believed to reflect less useful/maladaptive and more useful/adaptive pain coping strategies, respectively. In response to each item assessing a specific coping response, respondents are asked to rate how many days during the past week they used that strategy to cope with their pain. A higher score for each scale indicates more frequent use of the category of coping assessed by each scale. The CPCI-J has evidence supporting its reliability and validity in Japanese patients with chronic pain.^2^ The internal consistency coefficients for the CPCI-J scales in the current sample indicated adequate (at least 0.70) to excellent (over 0.90) reliability for most of the scales. The Seeking Social Support had marginal internal consistency (Cronbach's alpha = 0.66).

### 2.4. Data analysis

The demographic characteristics of the study participants were summarized by computing descriptive statistics. Next, we evaluated the factor structure of the PSOCQ-J using a confirmatory factor analysis (CFA). The model fit was evaluated with following fit indices: Chi-square goodness of fit index (χ^2^; a nonsignificant result at a 0.05 threshold indicates good fit^28^), Normed Chi-square (χ^2^/*df*; a value below 2–3 indicates an acceptable fit^28^), root mean square error of approximation (RMSEA; a value below 0.08 indicates an acceptable fit^29^), Comparative Fit Index (CFI; a value more than 0.95 indicates an acceptable fit^16^), and Akaike Information Criterion (AIC; with a smaller value indicating a more parsimonious model fit^28^). Maximum likelihood estimation was used to estimate models in the CFA. Because responses were missing for items 6 (2% missing) and 18 (1% missing) of the PSOCQ-J, a full information maximum likelihood method was applied to account for missing data. Because previous studies reported substantial associations between the 3 factors except for Precontemplation, we compared the fit of a 4-factor model with 2-factor (combining the 3 factors) and 3-factor (combining the Action and Maintenance) models using the likelihood ratio test. A sample size of 200 cases is considered adequate for CFA analyses.^28^

We then computed Cronbach's alpha coefficients, interscale correlation coefficients, and ICC coefficients to evaluate the internal consistency, between scale associations, and test–retest stability of the PSOCQ-J scales, respectively. We also performed independent *t* tests to determine whether there were sex differences in the PSOCQ-J scales. Next, to evaluate the construct validity of the PSOCQ-J scales, we computed the Pearson correlation coefficients between the PSOCQ-J scales and the other study measures. Also, we compared the means of the PSOCQ scales between participants participating or not participating in exercise treatment, and using or not using analgesic medication.

We used the criterion of *P* < 0.05 to determine statistical significance. Each of the validity criterion measures had very few missing values (range: 0%–1%). Thus, we used pairwise deletion to compute the validity correlation coefficients. We used Mplus version 8.0.^33^ for the CFA analyses and the statistical package R version 3.2.4.^39^ for the other analyses.

## 3. Results

### 3.1. Demographic characteristics of participants

A total of 201 participants provided data for this study. Their mean age ± SD was 61.32 ± 13.93 years (range: 26–84), and their mean pain duration was 91.99 ± 110.21 months (range: 3–948; 0.25–79.00 years). Most participants were women (66%), were married (73%), had at least high school education (90%), and had a job or were homemakers (59%). The most common primary pain location was low back (28%). About one-third of the participants attended an exercise treatment. Seventy-one participants (35%) provided responses to the PSOCQ-J 2 occasions, with an average of 35.72 ± 19.34 days (range 7–77 days) between assessments. Additional descriptive details regarding the study participants and study measures are presented in Tables 1 and 2.

**Table 1:**
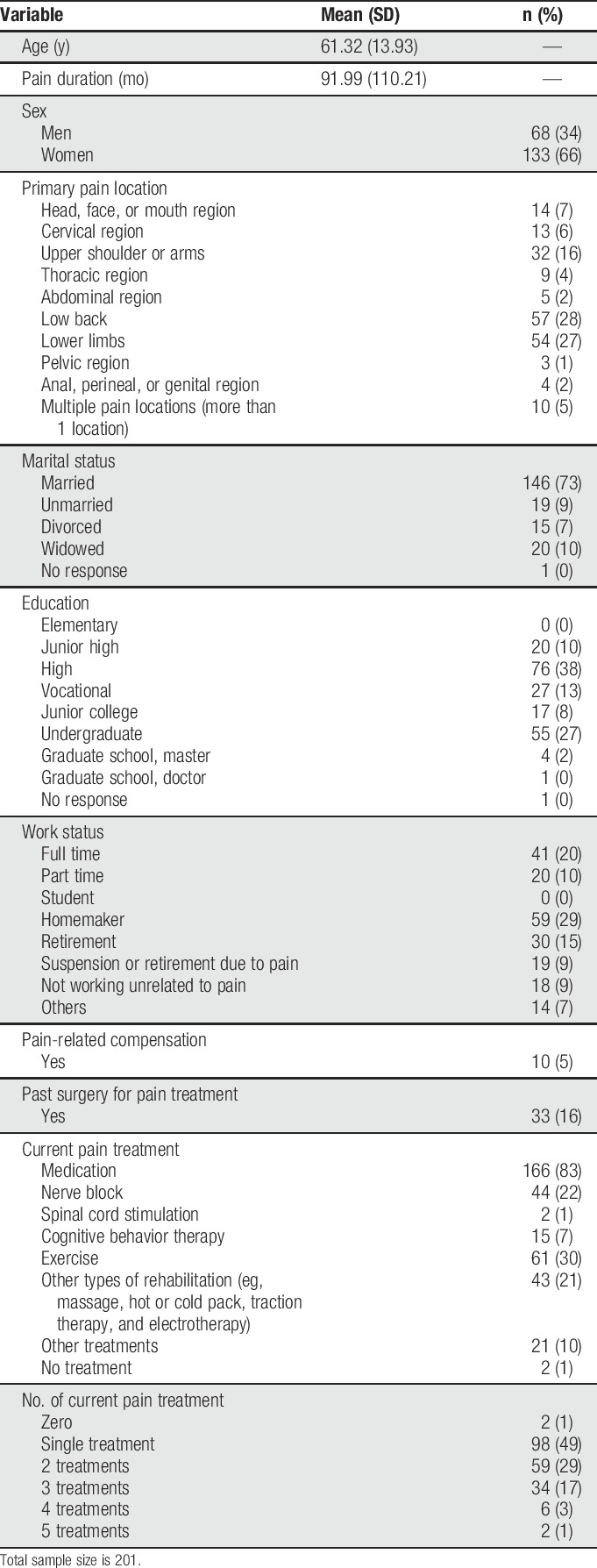
Demographic characteristics of the study participants.

**Table 2:**
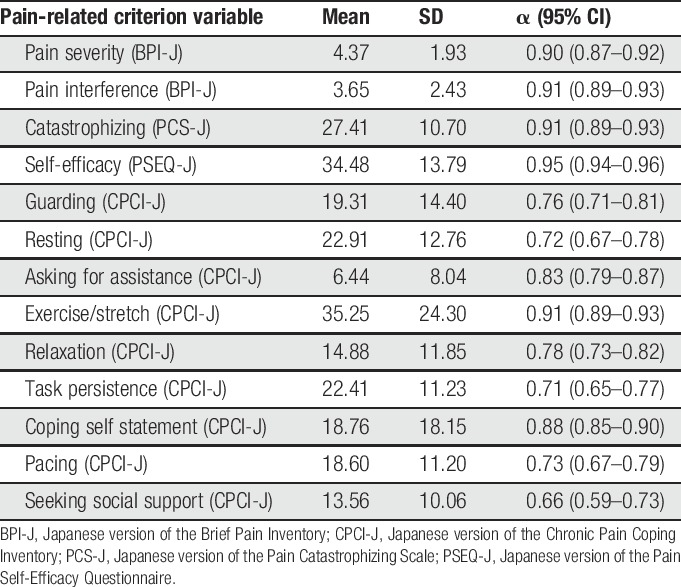
Mean values, SDs, Cronbach's alpha coefficients of the pain-related measures.

### 3.2. Factor structure of the Japanese version of the Pain Stage of Change Questionnaire

**Figure 1.**
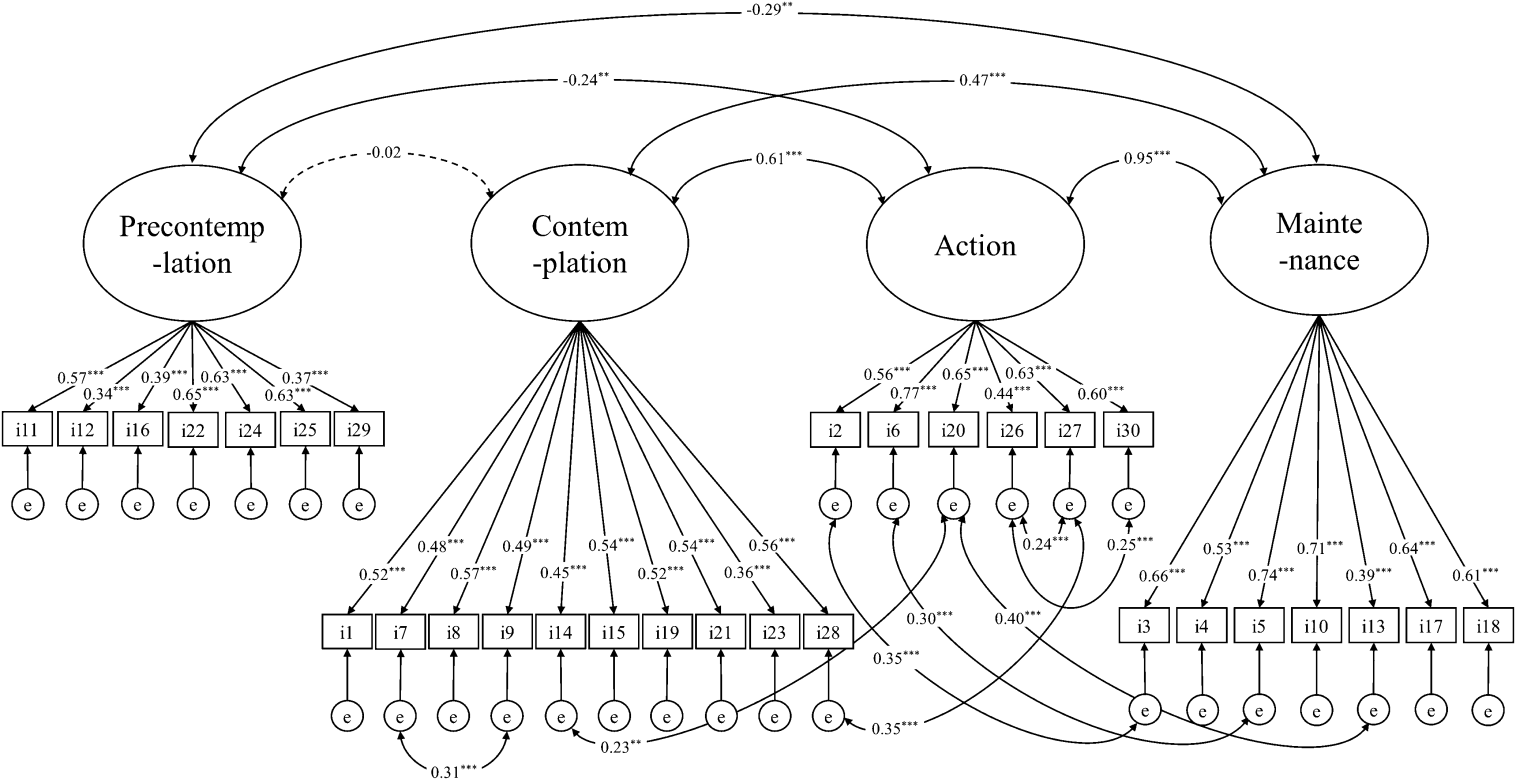
Factor structure of the Japanese version of the Pain Stage of Change Questionnaire. *P < 0.05, **P < 0.01, ***P < 0.001. The “i” represents the item. Thus, for example, the “i1” represents item 1 of the Japanese version of the Pain Stage of Change Questionnaire. The “e” represents error. We reported standardized parameter estimate values.

Model fit statistics for the first 4-factor model did not indicate sufficient fit (χ^2^_(399)_ = 972.23, *P* < 0.001, χ^2^/*df* = 2.44, RMSEA = 0.09, CFI = 0.71, and AIC = 15581.91). To improve this model, we allowed 8 error covariances between items with modification indices above 10. We chose these error covariances with reference to not only modification index but also overlap of phrasing and meaning (ie, the error variances of items that seem to assess similar aspects of readiness were allowed to correlate with one another). The second CFA showed acceptable fit (χ^2^_(391)_ = 812.74, *P* < 0.001, χ^2^/*df* = 2.08, RMSEA = 0.07, CFI = 0.78, and AIC = 15438.41). Item loadings of the second model ranged 0.34 to 0.77 (Fig. 1).

The CFA of the 2-factor model with the same error covariances showed poor fit (χ^2^_(396)_ = 966.96, *P* < 0.001, χ^2^/*df* = 2.44, RMSEA = 0.09, CFI = 0.71, and AIC = 15909.67), but the CFA of the 3-factor model using the same error covariances had acceptable fit (χ^2^_(394)_ = 824.53, *P* < 0.001, χ^2^/*df* = 2.11, RMSEA = 0.07, CFI = 0.78, and AIC = 15444.20). However, the results of the likelihood ratio test suggested significant deterioration when adopting either the 2-factor (χ^2^_(5)_ = 154.23, *P* < 0.001) or 3-factor models (χ^2^_(3)_ = 11.79, *P* < 0.01). Thus the evidence supported the 4-factor model over the other 2 models.

### 3.3. Reliability and interscale correlations of the Japanese version of the Pain Stage of Change Questionnaire scales

All the Cronbach's alpha coefficients for the PSOCQ-J scales exceeded 0.70 (range 0.72–0.80; Table 3), indicating at least adequate internal consistency. The ICCs between 2 measurement points showed good stability over time (range 0.62–0.71; Table 3). No significant sex differences were observed for PSOCQ-J scales. Moreover, the interscale correlations were consistent with the study hypotheses for the most part, except that we found an unpredicted significant positive association between the Contemplation and Maintenance scales (Table 4).

**Table 3:**
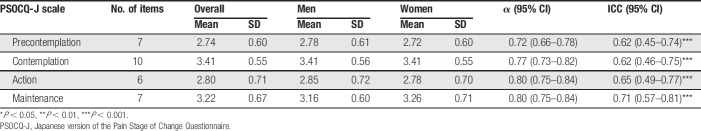
Mean values, SDs, Cronbach's alphas, and intraclass correlations (ICC) of the PSOCQ-J scales.

**Table 4:**
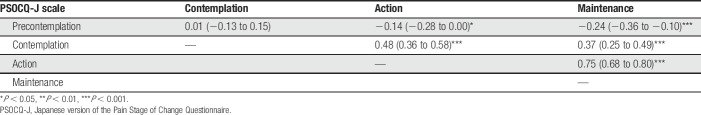
Interscale correlations of the PSOCQ-J scales.

### 3.4. Correlations between the Japanese version of the Pain Stage of Change Questionnaire scales and pain-related measures

The zero-order correlations between the 4 PSOCQ-J scales and the validity variables assessing pain severity, pain interference, self-efficacy, and catastrophizing are presented in Table 5. As can be seen, and as hypothesized, the Precontemplation scale had significant weak to moderate positive correlations with the measures of pain severity, pain interference, and catastrophizing, whereas it had a weak negative correlation with the self-efficacy measure. The Maintenance scale had weak negative correlations with the measures assessing pain severity, pain interference, and catastrophizing, whereas it had a significant weak positive correlation with the self-efficacy measure. The Action scale had a weak positive correlation with the self-efficacy measure and a weak negative correlation with the catastrophizing measure. The Contemplation scale had a weak positive correlation with the pain interference measure.

**Table 5:**
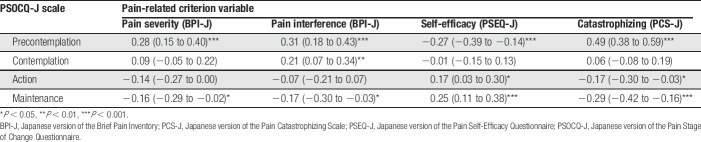
Pearson correlations between PSOCQ-J scales and measures assessing pain severity, pain interference, self-efficacy, and catastrophizing.

The zero-order correlations between the PSOCQ-J scales and CPCI-J scales are presented in Table 6. The Precontemplation scale showed significant weak positive correlations with the 3 maladaptive pain coping scales. The Action and Maintenance scales had weak to moderate positive correlations with the 6 adaptive pain coping scales, except for the nonsignificant correlation between the Maintenance scale and the Seeking Social Support scale. The Contemplation scale had weak positive correlations with the 9 CPCI-J scales.

**Table 6:**
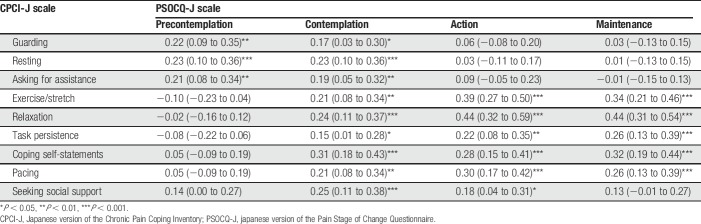
Pearson correlations between PSOCQ-J and CPCI-J scales.

### 3.5. Comparison of scores of the Japanese version of the Pain Stage of Change Questionnaire scales with respect to current treatments

Mean values and SDs of the PSOCQ scales between the participants who were participating or not participating in exercise treatment, and between the participants taking analgesic medications or not, are presented in Table 7. The independent *t* tests revealed significant differences in the mean values of the PSOCQ scale as a function of treatment. Specifically, the participants who were participating in exercise treatment scored lower on Precontemplation (*t*_(199)_ = 2.31, *P* = 0.02), and higher on Contemplation (*t*_(199)_ = −4.12, *P* < 0.001), Action (*t*_(194)_ = −5.61, *P* < 0.001), and Maintenance (*t*_(196)_ = −5.26, *P* < 0.001) than the participants who were not. No significant differences in the mean values of the PSOCQ scales were found between the participants who were taking analgesic medications or not.

**Table 7:**
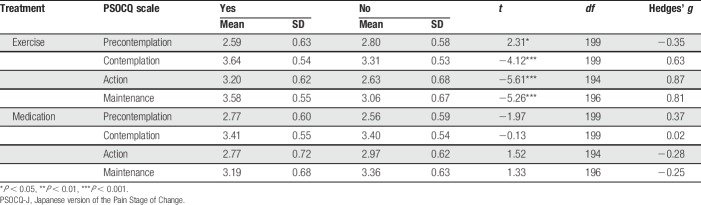
Mean values and SDs of PSOCQ-J scales based on the current treatment.

## 4. Discussion

Here, we sought to translate and evaluate the psychometric properties of the PSOCQ-J. The findings supported a 4-factor structure, and all 4 scales of the PSOCQ-J evidenced good internal consistencies and test–retest reliabilities. In addition, the results supported the hypothesized pattern of between scale associations, except for a substantial positive association between the Contemplation and Maintenance scales. The study hypotheses with respect to construct validity of the PSOCQ-J scales were also supported.

The 4-factor structure, with items loading on the Precontemplation, Contemplation, Action, and Maintenance factors, is consistent with the results of previous research in samples of individuals with chronic pain from diverse clinical and cultural settings and using different language versions of the measure.^13,19,26,31,32^ The present results also supported the 4-factor model over the 2-factor and 3-factor models. Thus, the findings provide support for cross-cultural validity of the 4 readiness domains hypothesized by the PRC model.^24^

On the other hand, the substantial associations between the PSOCQ-J scales cast some doubt on the “stage” concept underlying the PRC model, that is, the idea that people necessarily move from one stage to the next, as they become more ready to self-manage pain. Previous studies have also reported significant associations between PSOCQ scales.^6,13,19,26,31,41,42^ This suggests the possibility that readiness to change might be better viewed as a continuum than as discrete stages.^18^ In addition, the strong association found between the Contemplation and Maintenance scales in the current sample cast some doubt on the utility of viewing these as entirely distinct constructs. Thus, participants who are considering the usefulness of pain self-management (ie, who score high on the Contemplation scale) may also be using a number of self-management approaches to manage their pain (ie, they may also score high on the Maintenance scale).

The internal consistency and test–retest reliability results supported the reliability of the PSOCQ-J scales. That said, the stabilities of the PSOCQ-J scales were somewhat lower than those reported in previous studies.^26,32^ This difference may be due, at least in part, to the longer period between assessments in the current study, relative to other studies, combined with the expectation that readiness to self-manage pain is believed to be influenced by many factors, that is, readiness to self-manage pain may be more a state than a trait.^18,24^

The findings also support the construct validity of the PSOCQ-J. The 4 scales generally evidenced the patterns of associations with other pain-related measures and pain treatments that were hypothesized a priori. As expected, the Precontemplation and Maintenance scales evidenced patterns of associations suggest that the former may be a less useful/maladaptive cognitive and behavioral state, whereas the latter may reflect a more useful/adaptive one.

There are a number of limitations to this study that should be considered when interpreting the results. First, the sample was one of convenience, made up of consecutive patients seen in a number of specific clinics who agreed to participate in the study. The average age of participants was relatively high, most of the participants were women, and many were not receiving training in treatments designed to enhance their ability to self-manage pain. Thus, the extent to which the findings would replicate in other samples of individuals with chronic pain from Japan remains unknown. Additional research with other samples is needed to determine which of the findings replicate. Second, this study used a cross-sectional design to evaluate the validity of the PSOCQ-J. Thus, we were not able to evaluate the extent to which the PSOCQ-J scales are sensitive to treatments designed to enhance readiness to self-manage pain, nor were we able to determine the causal influence of changes in the PSOCQ-J scales and subsequent changes in important outcomes. Longitudinal and clinical trial research is needed to evaluate these aspects of the measure's validity. Third, we did not collect any data that would allow us to determine, separately from the PSOCQ-J, what readiness stage the participants might be in. For example, patients who are just beginning an exercise program might be expected to be at a lower readiness stage for pain self-management than patients who have been exercising regularly for many months.

Despite the study's limitations, the findings provide important information regarding the reliability and validity of the PSOCQ-J. We found that the measure has a 4-factor structure, and provides reliable and valid measures of Precontemplation, Contemplation, Action, and Maintenance. Thus, the findings support the use of the PSOCQ-J to evaluate the role that readiness to self-manage pain may have in adjustment to chronic pain in Japanese chronic pain populations.

## Disclosures

The authors have no conflict of interest to declare.

This study was presented at the 7th Asian Pain Symposium on October 27, 2017, as a poster presentation.
